# 122 PTSD Symptom Clusters as Predictors of Pain Interference in Burn Survivors

**DOI:** 10.1093/jbcr/irac012.124

**Published:** 2022-03-23

**Authors:** Shelley A Wiechman, Arjun Bhalla, Alyssa M Bamer, Gretchen J Carrougher, Barclay T Stewart, Nicole S Gibran, Jeffrey C Schneider, Christina Temes, Frederick J J Stoddard, Kimberly Roaten

**Affiliations:** University of Washington, Seattle, Washington; University of Washington, Seattle, Washington; University of Washington, Golden, Colorado; Department of Surgery, The University of Washington, Seattle, Washington; University of Washington, Seattle, Washington; University of Washington, Wellfleet, Massachusetts; , Massachusetts; Massachusetts General Hospital/Spaulding Rehabilitation Hospital, Boston, Massachusetts; Harvard Medical School, Boston, Massachusetts; UT Southwestern Medical Center, Dallas, Texas

## Abstract

**Introduction:**

Individuals who experience burns are at higher risk of developing post-traumatic stress disorder (PTSD) and chronic pain. There exists a synergistic relationship between PTSD and chronic pain in burn survivors. Theories exist about how aspects of each condition may perpetuate one another, or share underlying mechanisms. Both of these conditions are of relevance to pain-related disability. We sought to examine the role of individual PTSD symptom clusters as predictors of pain interference. We hypothesized that the hyperarousal and emotional numbing symptom clusters would be predictive of pain interference, even when accounting for the other two PTSD symptom clusters, pain intensity, and other covariates (burn size, hospital length of stay, age and gender).

**Methods:**

Data were analyzed from the Burn Model System National Database. Inclusion criteria required participants to have a moderate to severe burn injury that required surgery for wound closure. Patient-reported outcome data: PTSD Checklist - Civilian, PROMIS-Pain Interference Short Form 4a, and a 0-10 average Pain Intensity item were analyzed at 6-months after injury. Hierarchical linear regression models were fit to examine the impact of PTSD symptom clusters on pain interference over and above that of pain intensity, and standardized betas were calculated (*B*).

**Results:**

A total of 439 adult participants had complete responses on the measures of interest (e.g. PTSD symptoms, PROMIS-Pain Interference, and Pain Intensity) and were included in the analysis. Mean age, percent total body surface area burned, and hospital length of stay were 47 years, 18%, and 27 days, respectively. 69% were male and 82% were Caucasian. Results of a linear regression found that hyperarousal (*B* = .10, *p* = .03) and emotional numbing (*B* = .13, *p* = .01) PTSD symptom clusters were each significant predictors of pain-related disability, even when accounting for pain intensity (*B* = .64, *p* < .001). The covariates age, gender, days until discharge, and TBSA were all nonsignificant. The model accounted for 61% of the variance associated with pain-related disability.

**Conclusions:**

Results highlight the importance of the emotional numbing and hyperarousal PTSD symptom clusters in explaining pain interference. Future evaluations parsing out the longitudinal relationships (i.e., beyond 6-months postburn) between PTSD symptom clusters, pain intensity, and pain interference, as well as evaluating other underlying mechanisms, are warranted.

